# Modeling, Validation, and Performance of Two Tandem Cylinder Piezoelectric Energy Harvesters in Water Flow

**DOI:** 10.3390/mi12080872

**Published:** 2021-07-25

**Authors:** Rujun Song, Chengwei Hou, Chongqiu Yang, Xianhai Yang, Qianjian Guo, Xiaobiao Shan

**Affiliations:** 1 School of Mechanical Engineering, Shandong University of Technology, Zibo 255049, China; songrujun@sdut.edu.cn (R.S.); hcwking@163.com (C.H.); yangcq@sdut.edu.cn (C.Y.); yxh@sdut.edu.cn (X.Y.); 2 Shenzhen Research Institute of City University of Hong Kong, Shenzhen 518057, China; 3 School of Mechatronics Engineering, Harbin Institute of Technology, Harbin 150001, China

**Keywords:** piezoelectric energy harvester, tandem, energy harvesting, vortex-induced vibration, flowing water

## Abstract

This paper studies a novel enhanced energy-harvesting method to harvest water flow-induced vibration with a tandem arrangement of two piezoelectric energy harvesters (PEHs) in the direction of flowing water, through simulation modeling and experimental validation. A mathematical model is established by two individual-equivalent single-degree-of-freedom models, coupled with the hydrodynamic force obtained by computational fluid dynamics. Through the simulation analysis, the variation rules of vibration frequency, vibration amplitude, power generation and the distribution of flow field are obtained. And experimental tests are performed to verify the numerical calculation. The experimental and simulation results show that the upstream piezoelectric energy harvester (UPEH) is excited by the vortex-induced vibration, and the maximum value of performance is achieved when the UPEH and the vibration are resonant. As the vortex falls off from the UPEH, the downstream piezoelectric energy harvester (DPEH) generates a responsive beat frequency vibration. Energy-harvesting performance of the DPEH is better than that of the UPEH, especially at high speed flows. The maximum output power of the DPEH (371.7 μW) is 2.56 times of that of the UPEH (145.4 μW), at a specific spacing between the UPEN and the DPEH. Thereupon, the total output power of the two tandem piezoelectric energy harvester systems is significantly greater than that of the common single PEH, which provides a good foreground for further exploration of multiple piezoelectric energy harvesters system.

## 1. Introduction

Global environmental and energy policies stress the need to increase the share of renewable resources and to enhance the efficiency of energy conversion plants and commit to developing advanced solutions for power production [1,2,3,4]. In addition to the conversion of biogas to electricity, which has attracted many scholars, the use of piezoelectric materials to convert kinetic energy in the environment into electrical energy has also become a hot spot in electrical energy conversion. Fluid kinetic energy is one of the most important clean energy resources and is widely distributed. It is of great value to convert fluid kinetic energy to electricity for human use. There are several efficient methods for energy transformations, such as turbines for hydro-plants, windmills for wind farms, and so on. Both turbines and windmills are suitable for large-scale power generation, which is inconvenient for portable, wearable, low-energy device. The piezoelectric effect can be applied for energy harvesting [5,6,7] and ultrasonic transduction [8,9,10,11]. Especially, piezoelectric energy harvesters (PEHs) have been widely used for energy harvesting from ambient vibration [12,13,14,15,16]. Hence, the piezoelectric conversion mechanism can be used to capture energy from fluid kinetic energy, which has attracted much attention among scholars [17,18,19,20,21,22].

Flow-induced vibration (FIV) and biogas are renewable and alternative sources of energy [23,24,25]. FIV is a common natural phenomenon, such as vortex-induced vibration of smokestacks and power transmission lines, fluttering flags in airflow, and the flow-induced vibration of seaweed and submarine cables in ocean currents. Biogas and biomass gas (syngas) have been used to feed solid oxide fuel cells [26,27]. Bochentyn et al. [28] proposed using praseodymium and samarium co-doped with ceria as an anode catalyst for DIR-SOFC fueled by biogas, and investigated the catalytic performance. De Lorenzo et al. [29,30] investigated the electrical and thermal analysis of an intermediate-temperature solid oxide fuel cell system fed by biogas. Energy harvesting from the above FIV phenomenon will be an effective method for converting fluid kinetic energy to electric energy, which can be regarded as wake-induced vibration (WIV) [31,32,33,34], vortex-induced vibration [35,36,37,38,39,40], fluttering [41,42,43,44] and galloping [45,46,47,48], etc. Particularly, PEHs based on the WIV have been extensively investigated in the past decade years. Taylor et al. [49] and Allen et al. [50] first proposed an eel-shaped PEH, based on the WIV to convert water kinetic energy into electric energy. Akaydin et al. [51] studied an airflow PEH by WIV and found that the maximum power output could be obtained when the tip of the piezoelectric beam was located downstream about twice the cylinders’ diameter. Weinstein et al. [52] proposed that PEH caused by the awakening of the upstream cylinder, achieving output power of 3 mW at air velocity of 5 m/s. Li et al. [53] conducted a numerical study on the feasibility of the blunt body obtaining energy from steady state flow when *Re* = 100 and found that the performance was closely related to the local vortex dissipation and pressure gradient of large vortex in the wake near the PEH. Yu et al. [54] proposed four structures of cylindrical and PEH membranes. The beat dynamics of PEH membranes were extensively studied, which is helpful to comprehensively understand the energy collection process of elasticity, kinetic energy, and continuous energy transfer. It can be inferred that an enhanced performance of PEH can be achieved by using the WIV phenomenon.

Inspired by the WIV, some researchers have proposed a combined piezoelectric energy harvesting system to improve space utilization and output power performance [55]. Li et al. [56] investigated the wake effect of the two tandem cylinders and found that the aerodynamic forces of the two cylinders were largely determined by the spacing ratio between them. Abdelkefi et al. [57] studied two tandem PEHs in airflow. It was found that when the distance between two PEHs exceeds a critical value, wake galloping of the upstream PEH would occur, and this wake galloping can improve the average output power of the total energy harvesting system. Hu et al. [58] evaluated the performance of twin, adjacent PEHs, based on mutual interference, under wind excitation, and found that the total output power of the two wind energy harvesters could achieve up to 2.2 times that of two single harvesters at the optimal relative position. All above works indicate that the PEHs with tandem arrangement can improve the energy-harvesting capacity of the overall system. Therefore, Shan et al. [59] proposed an energy-harvesting system with two identical piezoelectric energy harvesters in a tandem configuration and investigated the energy-harvesting performance by experimentation. However, the vibration response mechanism of fluid–structure interaction and the relationship between the output power of the harvester and flow field distribution were not studied. Hence, this work proposes a simulation method to study the performance of two tandem cylinder piezoelectric energy harvesters thoroughly and systematically. The validity of the proposed simulation method is verified through the experimental results. Through the simulation, the vibration response and energy-harvesting performance under wake excitation, including the variation rules of vibration frequency, vibration amplitude, power generation, and the distribution of flow field, can be easily obtained.

## 2. Physical Model and Simulation Method

**Figure 1:**
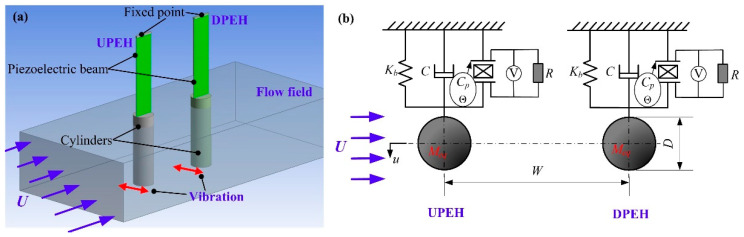
Schematic diagram of two tandem PEHs system: (**a**) three-dimensional system; (**b**) equivalent model of two tandem PEHs.

Figure 1 shows the physical model of a two tandem PEHs system. Each PEH consisted of a piezoelectric beam and a cylinder. The piezoelectric beam was fixed on the upper end and consisted of a piezoelectric layer (PZT-5H) and a substrate layer. The cylinder was attached to the free end and vertically immersed into incoming water. *U* is the water velocity. The water flowed over the cylinders and stimulated the PEHs to vibrate periodically. The energy harvesting system can be equivalent into two tandem independent single degree of freedom PEHs with separation spacing *W*. As shown in Figure 1, *u* is the vibration displacement of the PEH. *M_eq_*, *K_b_* are the equivalent mass and equivalent stiffness, respectively. *C* is the damping coefficient of the single PEH. *C_p_* is the equivalent capacitance of the PEH. Θ is the electromechanical coupling coefficient of mechanical vibration and electrical output. *V* is the generated voltage across the electrical load resistance *R*.

According to the equivalent model in Figure 1b, each of the two PEHs can be described as an individual-equivalent single-degree-of-freedom model, written as

(1) $$\left\{ \begin{array}{l} M_{eq}\ddot{u}+C\dot{u}+K_{b}u+\Theta V=F_{f}\\ \frac{V}{R}+C_{p}\dot{V}-\Theta \dot{u}=0 \end{array}\right.$$

where, *F_f_* is the flow-induced force, which should be obtained by the computational fluid dynamics (CFD) method (Fluent^®^ software) due to the complex interactions between the two tandem cylinders in water. *C* is the damping equaling to 2*ω_n_ξ*, and the *ξ* is the damping ratio and can be obtained by free decay oscillation method in experimental text. The second equation is based on the Kirchhoff’s law, which express that the sum of all the currents entering a node is equal to the sum of all the currents leaving the node. A key point of the measurement in the experiment is that the cylinder must be immersed into water when the free decay oscillation occurs. As a result, the added fluid damping of cylinder must be taken into account.

**Figure 2:**
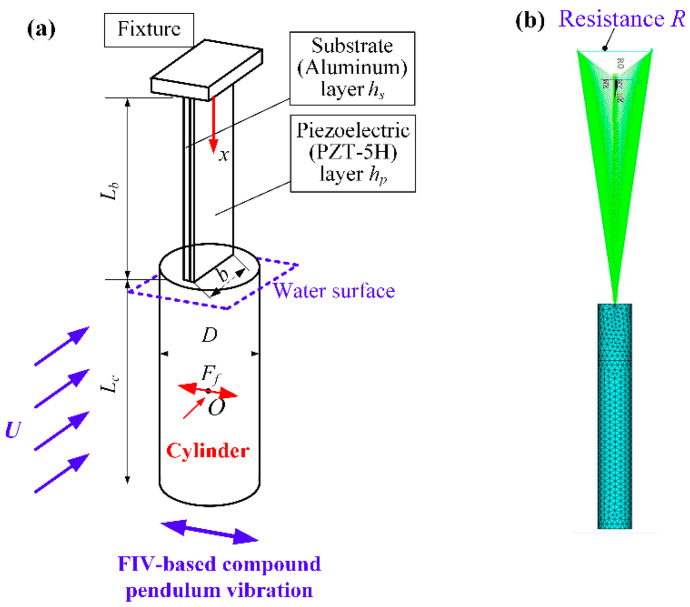
Physical model of the individual PEH: (**a**) Dimension diagram, (**b**) Finite element analytical model.

Figure 2a illuminates the schematic of the individual PEH. The length of piezoelectric beam and cylinder are *L_b_*, *L_c_*. The thickness of piezoelectric and substrate layer are *h_p_*, *h_s_*, respectively. The width of piezoelectric beam is *b*. The depth of immersion into water flow of the PEH is *L_f_*. Point *O* is the center of flow-induced force action on cylinder. Figure 2b shows the finite element analytical model of the PEH. Elements SOLID5, SOLID45, and CIRCU94, in ANSYS^®^ 10, were used for finite element simulation of the piezoelectric layer, substrate lay, and load resistance circuit, respectively. The sign of the piezoelectric constant was applied for indicating the polarization direction of the PZT-5H. The node on the upper piezoelectric layer surface was named common node “1”, and the node on the lower surface was named common node “2”. Then the load resistance *R* can be connected between the common nodes “1” and “2”.

According to dimension of PEH in Figure 2a, the deflection *w_b_*(*x*, *t*) of the cantilever beam can be expressed as

(2) $$w_{b}(x,t)=\frac{F_{f}x^{2}}{6EI}(3L_{b}-x)+\frac{M_{Force}x^{2}}{2EI}\;\;x\leq L_{b}$$

where *EI* is the stiffness of piezoelectric cantilever beam, expressed as

(3) $$EI=b\left[\frac{E_{p}\left(h_{c}{}^{3}-h_{b}{}^{3}\right)+E_{s}\left(h_{b}{}^{3}-h_{a}{}^{3}\right)}{3}\right]$$

where, *h_a_*, *h_b_*, and *h_c_* are expressed as

(4) $$\begin{array}{l} h_{a}=-\frac{h_{s}}{2}-\frac{\left(h_{p}+h_{s}\right)E_{p}h_{p}}{2\left(E_{p}h_{p}+E_{s}h_{s}\right)}\\ h_{b}=\frac{h_{s}}{2}-\frac{\left(h_{p}+h_{s}\right)E_{p}h_{p}}{2\left(E_{p}h_{p}+E_{s}h_{s}\right)}\\ h_{c}=\frac{h_{s}}{2}+h_{p}-\frac{\left(h_{p}+h_{s}\right)E_{p}h_{p}}{2\left(E_{p}h_{p}+E_{s}h_{s}\right)} \end{array}$$

where, *E_p_*, *E_s_* are the Young modulus of the piezoelectric and substrate materials.

The moment *M_Force_* acts on the tail of cantilever beam is expressed as

(5) $$M_{Force}=F_{f}\left(L_{c}-\frac{L_{f}}{2}\right)$$

The displacement of terminal cylinder *w*(*x*, *t*) is

(6) $$w(x,t)=w_{b}(L_{b},t)+w_{b}^{\prime }(L_{b},t)(x-L_{b})\;L_{b}\leq x\leq L_{b}+L_{c}$$

where, $w_{b}^{\prime }$ is the first derivative of *x*. Then equivalent stiffness *K_b_* can be obtained by the following equation, using Hooke’s law,

(7) $$K_{b}=\frac{F_{f}}{w\left(L_{bo},t\right)}$$

where, *L_bo_* = *L_b_* + *L_c_* − *L_f_*/2, and the *K_b_* is obtained at last.

The total kinetic energy of the PEH (TPEH) is the sum of kinetic energy of the piezoelectric layer and substrate layer (*T_beam_*), kinetic energy of cylinder (*T_cylinder_*), and kinetic energy of its added fluid (*T_fluid_*), expressed as follow

(8) $$T_{PEH}=T_{beam}+T_{cylinder}+T_{fluid}$$

where,

(9) $$\begin{array}{l} T_{beam}=\frac{1}{2}\int_{V_{p}}\rho_{p}{\left[\frac{\partial w_{b}(x,t)}{\partial t}\right]}^{2}dV_{p}+\frac{1}{2}\int_{V_{s}}\rho_{s}{\left[\frac{\partial w_{b}(x,t)}{\partial t}\right]}^{2}dV_{s}\\ T_{cylinder}=\frac{1}{2}\int_{L_{b}}^{L_{b}+L_{c}}\frac{M_{c}}{L_{c}}{\left[\frac{\partial w(x,t)}{\partial t}\right]}^{2}dx\\ T_{fluid}=\frac{1}{2}\int_{L_{b}+L_{c}-L_{f}}^{L_{b}+L_{c}}\frac{M_{cf}}{L_{f}}{\left[\frac{\partial w(x,t)}{\partial t}\right]}^{2}dx \end{array}$$

where, *ρ_s_* is the density of substrate material, *ρ_p_* is the density of piezoelectric material, *M_c_* is the mass of cylinder, and *M_cf_* is the fluid added mass, written as

(10) $$M_{cf}=C_{M}\frac{D^{2}}{4}\pi L_{f}\rho_{f}$$

where *C_M_* is the additional mass coefficient, *C_M_* = 1.

Meanwhile, the kinetic energy TPEH can be re-expressed as

(11) $$T_{PEH}=\frac{1}{2}M_{eq}{\left[\dot{w}(L_{bo},t)\right]}^{2}$$

and the *M_eq_* can be obtained based on Equations (8) and (11). Thus the equivalent mass *M_eq_* can be obtained by the equation:

$$M_{eq}=\left[\begin{array}{c} \int_{V_{p}}\rho_{p}{\left[\frac{\partial w_{b}(x,t)}{\partial t}\right]}^{2}dV_{p}+\int_{V_{s}}\rho_{s}{\left[\frac{\partial w_{b}(x,t)}{\partial t}\right]}^{2}dV_{s}\\ +\int_{L_{b}}^{L_{b}+L_{c}}\frac{M_{c}}{L_{c}}{\left[\frac{\partial w(x,t)}{\partial t}\right]}^{2}dx+\int_{L_{b}+L_{c}-L_{f}}^{L_{b}+L_{c}}\frac{M_{cf}}{L_{f}}{\left[\frac{\partial w(x,t)}{\partial t}\right]}^{2}dx \end{array}\right]/{\left[\dot{w}(L_{bo},t)\right]}^{2}$$

The Θ of electromechanical coupling coefficient of the single PEH in energy harvesting system can be expressed as

(12) $$\Theta =\sqrt{\left(w_{noc}^{2}-w_{nsc}^{2}\right)M_{eq}C_{p}}$$

where, *ω_noc_* is open circuit resonance frequency, and *ω_nsc_* is short circuit resonance frequency, which will be obtained by *R*_0_ = 0 and *R*_0_→∞ in ANSYS^®^ software, respectively. Equivalent capacitance *C_p_* can be

(13) $$C_{p}=\frac{\varepsilon_{33}^{S}bL_{b}}{h_{p}}$$

where $\varepsilon_{33}^{S}$ is the dielectric constant of piezoelectric material.

Furthermore, the output power of the PEH can be expressed as

(14) $$P=\frac{1}{T}\int_{0}^{T}\frac{V^{2}\left(t\right)}{R}dt$$

where *T* is the alternating period of output voltage.

The simulation results can be obtained by Equations (1) and (14) using the User Defined Function (UDF) in Fluent^®^ software. The simulation process can be summarized as:

(1) Flow-induced force *F_f_* can be obtained by Fluent^®^ software at the cylinder position in flow field at time *t*.
(2) The vibration displacement *u*, vibration velocity $\dot{u}$, vibration acceleration $\ddot{u}$ of the PEH, and electrical parameters *V*(*t*), *P* can be obtained by both Equations (1) and (14).
(3) The cylinder position and computational grid will be updated based on the PEH displacement *u* in step 2.
(4) One step ∆*t* is finished and next step *t* + ∆*t* will proceed. The performances of two tandem PEHs can then be obtained at last.

**Figure 3:**
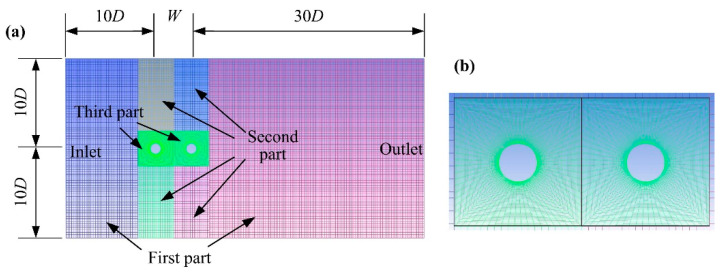
Schematic of the flow field grid of the tandem arrangement of energy harvesting system: (**a**) grid diagram of flow field; (**b**) partial grid diagram of third part.

Figure 3 illuminates the schematic of the flow field, including the whole grid arrangement and partial grid diagram of two tandem PEHs. The dynamic grid was applied in this CFD process. The domain of flow field grid was 20*D* × (40*D* + *W*), where *D* is the cylinder diameter and *W* is the spacing of the two tandem PEHs. The spacing of inlet to upstream piezoelectric energy harvester (UPEH) was 10 times that of *D*. The spacing of outlet to downstream piezoelectric energy harvester (DPEH) was 30 times that of *D*. In order to ensure the grid quality near the cylinders during the simulation process, the computational domain was divided into three parts, as shown in Figure 3a. The first part was the static grid. The second part was the dynamic grid, where the third part could move with the two tandem PEHs during the CFD process to reduce grid distortion, as shown in Figure 3b.

## 3. Experimental Setup and Simulation Verification

**Figure 4:**
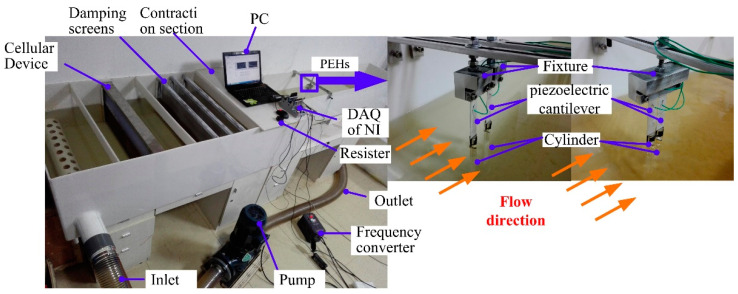
Energy harvesting experimental platform and prototypes of two tandem PEHs.

Figure 4 shows the experimental platform and the prototype setup. The experiment platform was made up of an open channel, fixtures, and measurement system. In order to obtain a steady flow, some sections in the open channel, such as the contraction section, damping screens, and a cellular device were set up to reduce stream turbulence. The speed of the water was controlled by a pump driven by a frequency converter. The fixtures were used to fix the two tandem PEHs, the spacing *W* was changed by relative position of the fixtures. The measurement system was composed of an adjustable resistor, a Data Acquisition system (DAQ) from National Instruments (NI), and a computer. The output voltage of the resistor was monitored by DAQ and displayed on the computer.

**Figure 5:**
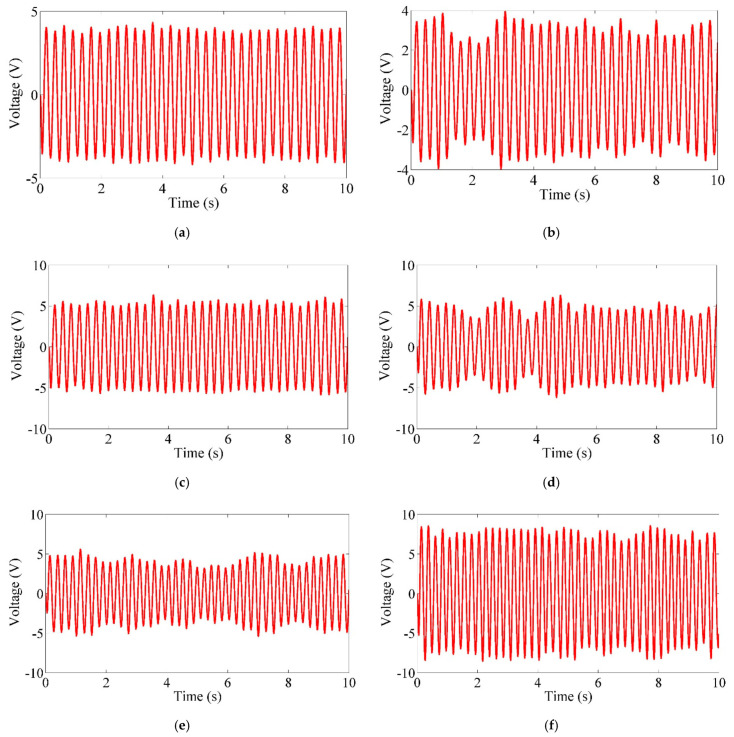
Output voltage of two tandem PEHs versus time for *W*/*D* = 3.3. (**a**) Output voltage of UPEH at *U* = 0.23 m/s; (**b**) Output voltage of DPEH at *U* = 0.23 m/s; (**c**) Output voltage of UPEH at *U* = 0.31 m/s; (**d**) Output voltage of DPEH at *U* = 0.31 m/s; (**e**) Output voltage of UPEH at *U* = 0.37 m/s; (**f**) Output voltage of DPEH at *U* = 0.37 m/s.

Figure 5 shows the output voltage evolution curve of the upstream and downstream PEHs with time when the spacing ratio *W*/*D* is 3.3 at the flow velocity of 0.23 m/s, 0.31 m/s and 0.37 m/s, respectively. The dimension and parameters of the two tandem PEHs are listed in Table 1. The comparison shows that when the flow velocity is 0.23 m/s, the output voltage of the upstream PEH was greater than that of the downstream PEH. At a flow velocity of 0.37 m/s, the output voltage of the upstream PEH was smaller than that of the downstream PEH. By comparing Figure 5a,c,e, it can be found that the output voltage of the upstream PEH at the velocity of 0.31 m/s is higher than that at the flow velocity of 0.23 m/s and 0.37 m/s, respectively. By comparing Figure 5b,d,f, it can be seen that the output voltage of the downstream PEH increases with the increase in velocity. It can be seen that under the tandem arrangement of the two PEHs, due to the strong coupling effect, the performance of the two PEHs will change greatly, especially the downstream one.

**Figure 6:**
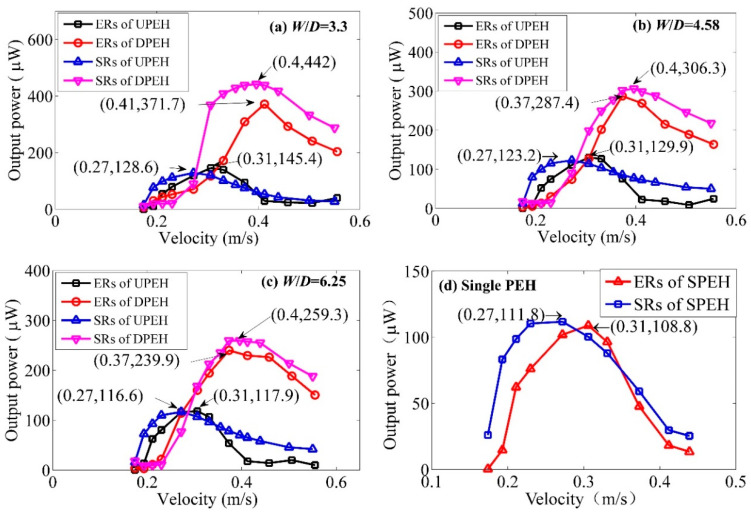
Output power of simulation results (SRs) and experimental results (ERs) of two tandem PEHs (**a**–**c**) and single PEH (**d**).

In order to research the energy-harvesting performance and verify the effectiveness of simulation method, Figure 6 illustrates the output power comparison between simulation and experimental results of the two tandem PEHs when the spacing ratio *W*/*D* is 3.3, 4.58, and 6.25, respectively. As can be seen from Figure 6a–c, when the spacing was smaller, the coupling effect was stronger, and the output power of the UPEH was slightly improved. With the increase in spacing ratio, the coupling effect was weakened and the energy harvesting performance of the UPEH decreased and gradually tended to resemble the performance of a single PEH. The experimental results showed that peak output power (145.4 μW of UPEH and 371.7 μW of DPEH) was generated at the velocities of 0.31 m/s and 0.41 m/s when *W/D* = 3.3, respectively. The maximum output power of DPEH was 2.56 times of that of the UPEH. When *W/D* = 6.25, the maximum output power of the UPEH was 117.9 μW and the maximum output power of the DPEH was 239.9 μW. It can be seen that the output performance of DPEH was better than that of UPEH, mainly occurred at the high flow velocity (>0.31 m/s). Especially, the output power of DPEH (371.7 μW) was 12.8 times that of UPEH (29.2 μW) when velocity was 0.41 m/s and *W/D* = 3.3. It can be summarized that the coupling effect of each PEH decreased with the increase in the spacing ratio of *W*/*D*. Meanwhile, the wake effect of the DPEH from the UPEH was gradually decreased, and the disturbance effect on UPEH from the DPEH was also weakened. It’s worth noting that the comparison between the theoretical results and the experimental data is better for high *W/D* values. This is because, in the experiment, the smaller the distance, the more serious the impact of the coupled vibration. The mutual disturbance is bound to cause disturbances in the flow field, aggravate wall disturbances, and change the depth of the cylinder’s water. In the theoretical analysis, the wall of the flow field is far enough away and the immersion depth of the two cylinders is assumed to be constant. This leads to smaller distances producing greater error between the simulation analysis results and the experimental results. The experimental results also show that the output power of UPEH and DPEH increased to the maximum first, and then decreased with the increase of the flow velocity, and the optimal velocity of the maximum power output could be obtained. In addition, the energy harvesting performance of a single piezoelectric energy harvester (SPEH) is given in Figure 6d. It can be noted that the simulation flow velocity corresponding to the maximum output power is lower than the experimental results. This is mainly because when the vortex-induced resonance occurs, the column immersion depth is slightly reduced, and the additional mass of water to the cylinder is slightly reduced too, but the immersion depth is assumed to be unchanged during the simulation, so that the natural frequency of the energy vibrator during simulation is slightly lower, which causes the flow velocity corresponding to the vortex-induced resonance to be slightly smaller than the experimental result. By comparing Figure 5a–c, it can be found that the power generation capacity of the UPEH and DPEH were greater than that of the SPEH. Therefore, it can be seen that the two tandem PEHs can improve the energy-harvesting performance of the system. What’s more, the overall results of the experiment and simulation are mild, and the error is within the allowable range. The main sources of error can be summarized as: in the experiment, the left and right swing caused a certain change in the depth of the cylinder’s water inlet. In the simulation, it is treated as quantitative, and some assumptions in the simplification process of the two-dimensional model will also have certain contrast errors. 

Therefore, the simulation results were consistent with the experimental results, as shown in Figure 6; the simulation method was verified.

## 4. Simulation Results and Discussion

**Figure 7:**
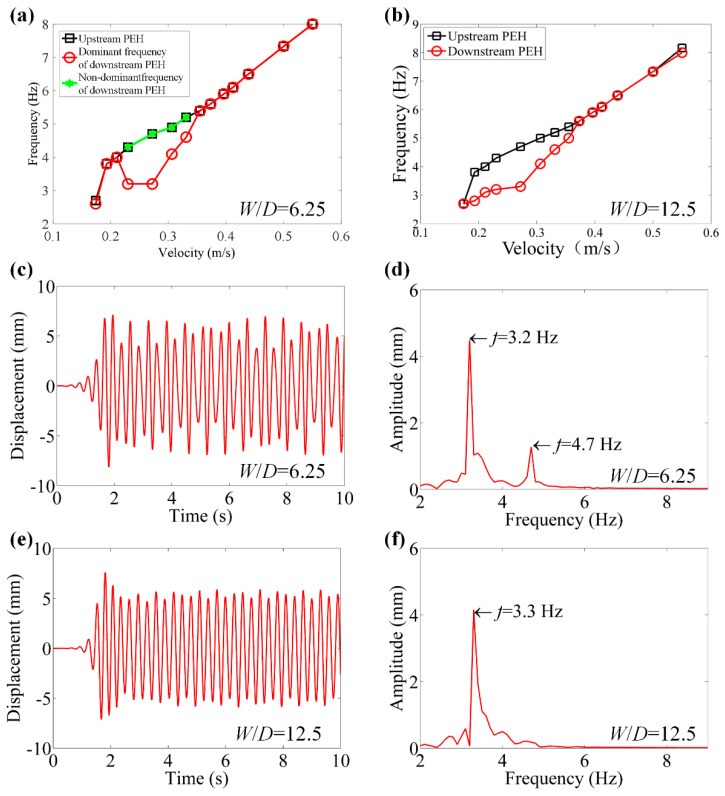
Vibration frequencies of the PEHs versus velocity when *W*/*D* = 6.25 and *W*/*D* = 12.5. (**a**) Vibration frequencies when *W*/*D* = 6.25. (**b**) Vibration frequencies when *W*/*D* = 12.25. (**c**) Vibration response when *W*/*D* = 6.25 and *U* = 0.272 m/s. (**d**) Fast Fourier transform (FFT) when *W*/*D* = 6.25 and *U* = 0.272 m/s. (**e**) Vibration response when *W*/*D* = 12.25 and *U* = 0.272 m/s. (**f**) FFT when *W*/*D* = 12.25 and *U* = 0.272 m/s.

In order to further investigate the performance of the two tandem PEHs, Figure 7a,b shows changes of vibration frequencies versus velocity. It can be concluded that frequency increases with velocity as a whole. However, as for the DPEH, the vibration performance is complicated. As shown in Figure 7a, the frequency increases with the increase of water velocity when water velocity (*U*) is less than 0.211 m/s, and then decreases with the increase of water velocity when 0.211 m/s < *U* < 0.23 m/s, and lastly increases with the increase of water velocity when *U* > 0.23 m/s. When the *W/D* = 12.5, the vibration frequency of DPEH was smaller than that of UPEH at low-speed flow, as shown in Figure 7b. Figure 7c–f are plotted for when *U* = 0.272 m/s. Due to the coupling effect of the vortex-induced vibration (VIV) itself and the wake-induced vibration (WIV) from the UPEH, there was inevitable unbalanced vibration in the DPEH, with small vibration frequency differences, such as the beat vibration signal, containing two frequency components of dominant frequency and non-dominant frequency when *W/D* = 6.25, which can be found that Figure 7c,d. When *W/D* = 12.5, the coupling effect becomes light, so that the frequency is unique as shown in Figure 7e,f.

**Figure 8:**
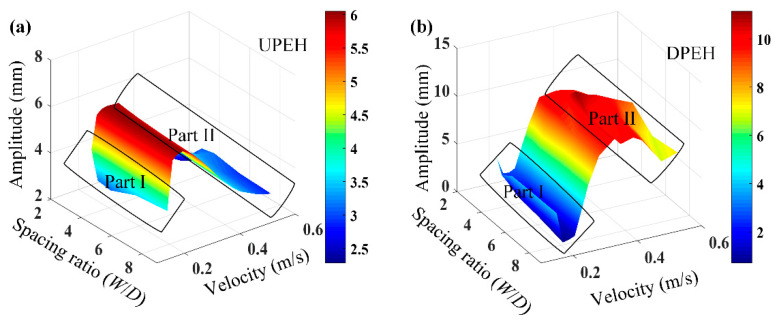
Comparison of vibration amplitudes of the upstream and downstream PEH: (**a**) UPEH, (**b**) DPEH.

In order to further analyze the vibration performance of the two tandem PEHs system, the vibration amplitudes of the upstream and downstream PEH with spacing ratio *W/D* and water speed were numerically discussed, specifically. As seen in Figure 8, Part I is the area of water velocity and Part II is the area of high-speed water velocity. As for the UPEH, the vibration amplitude was not mainly affected by the spacing ratio *W*/*D*, but rather by the water velocity. The vibration amplitude of the UPEH first increased and then decreased with the increase in velocity, and reached its maximum value when the velocity was about 0.27 m/s. As for the DPEH, the vibration amplitude first decreased to the minimum value when the velocity was small, as shown in Part I in Figure 8b. This phenomenon was attributed to the flow field transition of the shear layer around the DPEH; the vibration response was weakened during the transformation of the water flow from laminar to turbulent. With further increase in the water velocity, the amplitude of vibration on the DPEH increased to its maximum point, and then gradually decreased to a stable value, as shown in Figure 8b. Due to the excitation effect of upstream vortex shedding from the UPEH, the vibration response of DPEH was obviously greater than that of UPEH, especially at the high water speed, as shown in Part II in Figure 8a,b respectively.

**Figure 9:**
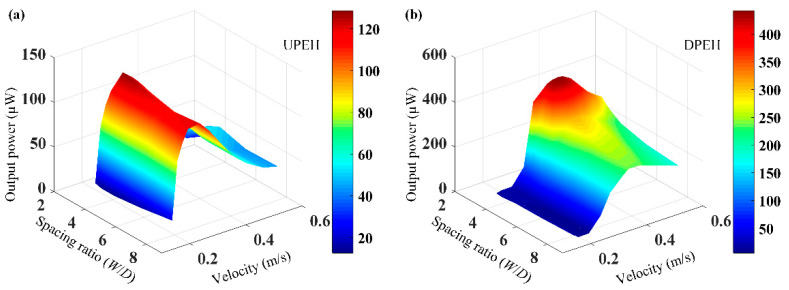
Energy harvesting performance of the two tandem PEHs versus spacing ratio *W*/*D* and water velocity: (**a**) UPEH, (**b**) DPEH.

In order to study the energy-harvesting performance of the system, the relationship among the output power of tandem PEHs, spacing ratio *W*/*D,* and water velocity are illustrated in Figure 9. It can be found that the output power of UPEH increased first and then decreased with the increase in water speed. The maximum output power was obtained at the speed of 0.27 m/s, at which point vortex-induced resonance occurred, combining with the vibration responses in Figure 8a. As for the DPEH in Figure 9b, the relationship between *W/D* and the energy-harvesting performance of the DPEH was the same as that of the UPEH. Comparing with the UPEH, the output power of the DPEH decreased slowly with the increase in velocity, due to wake stimulation from upstream, which was different from the performance of the UPEH. In addition, it can be concluded that in the range of low water velocity, the output power of the UPEH was higher than that of the DPEH, but in the range of high water velocity, the energy-harvesting performance of the DPEH was obviously better than that of the UPE,H due to wake stimulation by vortex shedding, which is in accordance with the vibration response results in Figure 8.

**Figure 10:**
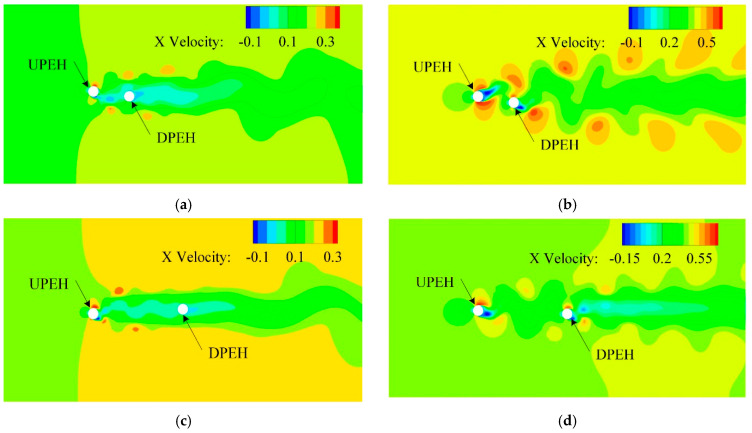
Flow field distribution of two tandem PEHs for various *W*/*D* and water velocities.(**a**) Flow field distribution at *W*/*D* = 3.33, *U* = 0.19 m/s; (**b**) Flow field distribution at *W*/*D* = 3.33, *U* = 0.41 m/s; (**c**) Flow field distribution at *W*/*D* = 3.33, *U* = 0.19 m/s; (**d**) Flow field distribution at *W*/*D* = 8.33, *U* = 0.41 m/s.

Further analyzing the coupling effect of the two tandem piezoelectric energy harvester, Figure 10 shows the velocity flow field contour in *x* direction with different spacing ratios and water velocities. It can be seen from Figure 10a,c that at a low flow speed (*U* = 0.19 m/s), a long low-speed flow region could be formed in the downstream after the water flowed through the upstream PEH (cylinder). At this time, the downstream PEH would be in the low-speed flow region. Therefore, the vibration response and power generation performance of the downstream PEH were weak at the low-speed water flow, as shown in Figure 9b. At the same time, the vibration response of the upstream PEH was similar to that of a single PEH at low speed flow, because the downstream PEH had a weak vibration response and its influence on the upstream PEH was negligible. According to Figure 10b,d, when the flow velocity was high (*U* = 0.41 m/s), the downstream PEH was located on the vortex path where the upstream PEH fallen off, and the surrounding vortices were relatively strong. Under stimulation of the vortex, the vibration response and power output performance of the downstream PEH would be greatly improved. In addition, as the vortex moved forward with the flow field, the vortex diffused and its turbulence strength decreased, so the vibration response and energy harvesting performance of the downstream PEH gradually decreased with the increase in spacing ratio.

## 5. Conclusions

In this paper, a tandem arrangement of piezoelectric energy harvesters was studied to scavenge the water flow vibration energy through simulation modeling and experimental validation. Through the simulation analysis, the variation rules of vibration frequency, vibration amplitude, power generation, and the distribution of flow field were obtained. The experiment results verify the accuracy of the simulation results. The effects of spacing ratio and water flow velocity on the vibration response and the output power of two tandem PEHs were studied numerically and experimentally. It could be concluded that the vibration response and energy-harvesting performance of the two tandem PEHs were enhanced due to the coupling effect induced by upstream vortex vibration. The vibration response of UPEH was vortex-induced vibration, and maximum power could be achieved at the resonant point of the PEHs and the vortex flow. When the spacing was small, the performance was enhanced due to enhancement of the DPEH. The vibration response of the DPEH was raised under the stimulation of the vortices shedding from the UPEH, especially under high-speed water flow; the energy-harvesting performance of the DPEH as better than that of the UPEH accordingly. However, when the flow speed was low, it was noticeable that the superiority of the DPEH energy harvesting was not remarkable and the power output of the UPEH was better than that of the DPEH. The results show that the energy-harvesting performance of the tandem PEHs in flowing water could be significantly improved, especially at the high speed flow region, which provides good support for further exploration of energy harvesting systems with multi-piezoelectric harvesters, parallel or serially arranged.

## Figures and Tables

**Table 1:** Dimension and material properties of the PEHs.

Parameters	Values
density of PZT-5H *ρ_p_* (kg/m^3^)	7386
density of aluminum layer *ρ_p_* (kg/m^3^)	2700
fluid density *ρ_p_* (kg/m^3^)	1000
Young modulus of PZT-5H *E_p_* (GPa)	59.77
Young modulus of aluminum layer *E_s_* (GPa)	71.7
length × width × thickness of piezoelectric layer (*L_b_* × *b* × *h_p_*) (mm)	80 × 20 × 0.2
length × width × thickness of substrate layer (*L_b_* × *b* × *h_s_*) (mm)	80 × 20 × 0.2
piezoelectric constant *e*_31_ (C/m^2^)	−13.74
dielectric constant $\varepsilon_{33}^{s}$ (F/m)	4.178 × 10^−8^
load resistance *R* (kΩ)	100
Cylinder diameter *D* (mm)	12
length of cylinder immersed in water *L_f_* (mm)	55
first mode damping ratio ζ	0.0499
open circuit resonance frequency *ω_noc_* (rad)	26.25
short circuit resonance frequency *ω_nsc_*(rad)	25.5
